# Hydrogen inhibits endometrial cancer growth via a ROS/NLRP3/caspase-1/GSDMD-mediated pyroptotic pathway

**DOI:** 10.1186/s12885-019-6491-6

**Published:** 2020-01-10

**Authors:** Ye Yang, Ping Yin Liu, Wei Bao, Song Jun Chen, Fang Su Wu, Ping Ya Zhu

**Affiliations:** 1 Department of Obstetrics and Gynecology, Shanghai General Hospital, Shanghai Jiao Tong University School of Medicine, 85 Wujin Road, Hongkou, Shanghai, 200080 People’s Republic of China; 2 Department of Obstetrics and Gynecology, Shanghai General Hospital of Nanjing Medical University, 85 Wujin Road, Hongkou, Shanghai, 200080 People’s Republic of China; 3 Department of Systems Biomedicine, Shanghai Jiaotong University, 800 Dongchuan Road, Biomedical Research Institute Building, Minhang, 200241 Shanghai, People’s Republic of China

**Keywords:** Hydrogen, Endometrial cancer, Pyroptosis, GSDMD, NLRP3

## Abstract

**Background:**

Pyroptosis belongs to a novel inflammatory programmed cell death pathway, with the possible prognosis of endometrial cancer related to the terminal protein GSDMD. Hydrogen exerts a biphasic effect on cancer by promoting tumor cell death and protecting normal cells, which might initiate GSDMD pathway-mediated pyroptosis.

**Methods:**

We performed immunohistochemical staining and western immunoblotting analysis to observe expression of NLRP3, caspase-1, and GSDMD in human and xenograft mice endometrial cancer tissue and cell lines. We investigated treatment with hydrogen could boost ROS accumulation in endometrial cancer cells by intracellular and mitochondrial sources. GSDMD shRNA lentivirus was used to transfect endometrial cancer cells to investigate the function of GSDMD protein in pyroptosis. Propidium iodide (PI) staining, TUNEL assay, measurement of lactate dehydrogenase (LDH) release and IL-1β ELISA were used to analysis pyroptosis between hydrogen-supplemented or normal culture medium. We conducted in vivo human endometrial tumor xenograft mice model to observe anti-tumor effect in hydrogen supplementation.

**Results:**

We observed overexpression of NLRP3, caspase-1, and GSDMD in human endometrial cancer and cell lines by IHC and western immunoblotting. Hydrogen pretreatment upregulated ROS and the expression of pyroptosis-related proteins, and increased the number of PI- and TUNEL-positive cells, as well as the release of LDH and IL-1β, however, GSDMD depletion reduced their release. We further demonstrated that hydrogen supplementation in mice was sufficient for the anti-tumor effect to inhibit xenograft volume and weight of endometrial tumors, as mice subjected to hydrogen-rich water displayed decreased radiance. Tumor tissue sections in the HRW groups presented moderate-to-strong positive expression of NLRP3, caspase-1 and GSDMD. Hydrogen attenuated tumor volume and weight in a xenograft mouse model though the pyroptotic pathway.

**Conclusions:**

This study extended our original analysis of the ability of hydrogen to stimulate NLRP3 inflammasome/GSDMD activation in pyroptosis and revealed possible mechanism (s) for improvement of anti-tumor effects in the clinical management of endometrial cancer.

## Background

Endometrial cancer is the most common gynecologic cancer. It is estimated by the American Cancer Society that there will be 61,880 new cases of endometrial cancer, and that 12,160 women will die of endometrial cancer in 2019 [1]. At present, the treatment of patients without fertility requirements is still based on surgery, and those who possess high risk factors also require radiotherapy or chemotherapy that kills cancer cells by augmenting reactive oxygen species (ROS)-mediated stress and activating apoptosis [2]. Although cancer-associated defects in apoptosis induction might occur, an “oncogene addiction” phenomenon might also arise and lead to therapeutic failure [3]. Therefore, other forms of cell death are being investigated to improve the prognosis of endometrial cancer. Since patients with endometrial cancer have elevated oxidative stress and systemic inflammatory conditions caused by metabolic syndrome, we hypothesized that inflammatory pathways were important as estrogen metabolism aspects of endometrial cancer, and that activation of the inflammation-mediated cell death pathway may facilitate the elimination of tumor cells.

Pyroptosis is an inflammatory programmed cell death pathway. It was termed “pyroptosis” as “Pyro” originates from the Greek word for fire, equals fever and inflammation for caspase-1 and IL-1β. “Ptosis” (silent “p”) origin from the Greek word “falling” means other forms of cell death [4]. The phenomenon of pyroptosis was first observed in macrophages infected with the mouse *Salmonella typhimurium* strain [5, 6]. Subsequent cytoplasmic cell swelling, lysis and vacuolization, membrane pore formation, DNA fragmentation, chromatin condensation, and inflammasome-mediated caspase-1 activation, as well as over- production of the proinflammatory cytokines IL-1β and IL-18, result in the release of cellular contents to the surrounding microenvironment [7], which then alarm and recruit neighboring cells to the location of infection.

Recent findings have revealed that the nucleotide-binding domain (NOD)-like receptor (NLR) family member pyrin domain-containing protein 3 (NLRP3) activates the inflammasome and can trigger pyroptosis [4, 8]. Key components of a functional NLRP3 inflammasome are NLRP3, the adaptor protein apoptosis associated speck-like protein containing ASC (a caspase recruitment domain, CARD), and the proinflammatory caspase-1 [9]. ROS/tumor necrosis factor (TNF-α)/nuclear factor-κB (NF-κB) signaling can then induce NLRP3 activation (Additional file 1) [10–15]. Upon this cellular stress, NLRP3 oligomerizes and presents clustered pyrin domains (PYD) for interaction with the PYD domain of ASC. CARDs of ASC then interact with the CARD of pro-caspase-1, which enables caspase-1 activation. Caspase-1 only participates in pyroptosis and does not mediate apoptosis, but caspase-1 exhibits two important roles: (1) to cleave off the suppressor C-terminal domain of a 53 kDa protein called gasdermin D (GSDMD) and liberate the pore-forming N-terminal domain of GSDMD, which then self-assembles to form pores in the plasma membrane; and (2) to convert the precursors of the proinflammatory cytokines IL-1β and IL-18 into their active forms, mature IL-1β and IL-18 [16], thus allowing the inflammatory process to be finally mediated as pyroptosis [9, 17, 18]. The human gasdermin gene family member GSDMD was recently identified as the key executor of pyroptosis [18], and the GSDMD protein requires an activator, such as LPS and nigericin, to initiate the death signal; however, these activators are limited to the laboratory and it has been proven difficult to translate them clinically. Thus, it is important to find safe and effective activators that can be translated into clinical applications.

**Fig. 1:**
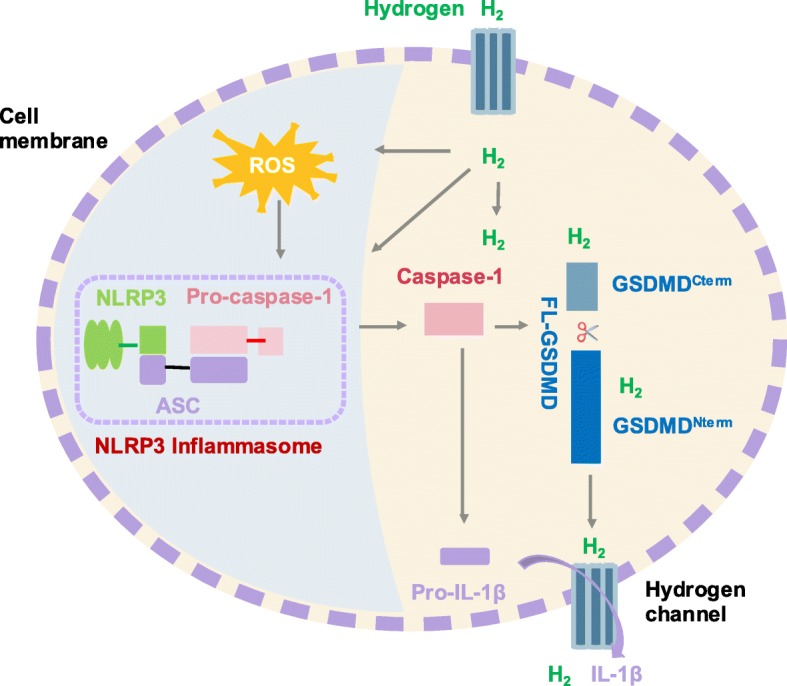
A proposed model depicting the possible mechanism underlying hydrogen/GSDMD- mediated cell pyroptosis in endometrial cancer. Hydrogen triggers the activation of ROS/NLPR3 signaling, which enables caspase-1 activation. GSDMD is cleaved off the suppressor C-terminal domain of the protein and liberates the pore-forming N-terminal domain of GSDMD, which then self-assembles to form pores in the plasma membrane that we call “hydrogen channels.” This then regulates the precursor of the proinflammatory cytokine IL-1β into its active form, mature IL-1β. These inflammatory genes constitute an inflammatory microenvironment in which to modulate cellular pyroptosis

Diatomic hydrogen (H_2_) exerts a biphasic effect of inhibiting tumor cell growth and protecting normal healthy cells. Biologic membranes are quite permeable to H_2,_ as they diffuse into the cytosol, mitochondria, and nucleus in relation to their distribution characteristics [19] (Fig.1). In contrast, most hydrophilic antioxidants cannot penetrate biomembranes and instead remain on the membrane surface. H_2_ can be used as an inert gas at body temperature or dissolved in water or saline at a concentration up to 0.8 mM (1.6 ppm) under atmospheric pressure to produce hydrogenated water or hydrogen-rich saline, or for producing hydrogen-generating nanomaterials [20]. In the past 10 years, H_2_ has been used to treat cancer and metabolic disease or to correct ischemia and reperfusion injury of various organs, taking advantage of its high bio-safety [20–22]. With respect to cancer, hydrogen can inhibit cellular activity, proliferation, invasion, and migration in a dose- and time-dependent manner; promote apoptosis in cervical cancer, breast cancer, cutaneous melanoma [23], ovarian cancer [24], lung cancer [25], colon cancer [26], thymic lymphoma [27], Ehrlich ascites tumor cells [28], oral squamous cell carcinoma, and fibrosarcoma cells [29]; reduce tumor volume and weight; and inhibit tumor growth in xenografted mice, thus prolonging the survival of tumor-bearing mice [23]. H_2_ also reduces renal toxicity in patients undergoing radiotherapy and chemotherapy [30]. Therefore, hydrogen therapy has received increasing attention and is now accepted as a promising therapeutic method.

Preliminary data from our lab indicated that hydrogen induced TNF/NF-κB and apoptotic pathways in endometrial cancer cells (Additional file 2). However, there are no extant reports on whether H_2_ can also activate pyroptosis or how it might function in endometrial cancer if apoptosis resistance caused by chemotherapy or radiotherapy occurs. It also remains unclear whether H_2_ inhibits tumor growth in endometrial cancer and through which signaling pathway it acts. Moreover we aimed to use mice model to address scientific objectives before clinical research. Therefore, we focus on these aspects in the present study.

## Methods

### Production of hydrogenated water and DMEM culture medium

Hydrogenated water was produced by H_2_ dissolved in water saturantly under 0.4 MPa pressure for 6 h with a concentration of 1.0 ppm produced by hydrogen water apparatus (provided by Shanghai Yi Quan Investment Limited Partnership Company, Shanghai, China). The hydrogenated water is contained of 400–1800 ppb nanoparticle, and a − 650 mV oxidation potential under this appropriate concentration, which does not easily evaporate or collapse in water, thus can stably supply hydrogen to cells and tissues better than hydrogen particles. The hydrogenated water was stored in a no dead volume bag made of aluminum at 4 °C under atmospheric pressure with 0.6 mmol/L of freshly prepared H_2_ every week before use.

Next we combined 28 ml of sterile hydrogenated water (1.0 ppm), 8 mL of 5X DMEM, 4 ml of 10% fetal bovine serum (FBS), 100 U/mL of penicillin G, 100 μg/mL of streptomycin to prepare the hydrogenated DMEM culture medium (H-CM) specially, which contained 0.7 ppm of diatomic hydrogen (H_2_) in this solution and was placed into a sterile centrifuge tube. The medium was freshly prepared every day and stored at 4 °C under atmospheric pressure before use. For the concentration measurements of dissolved molecular hydrogen in PBS, we used the hydrogen concentration detector (UNISENSE Danish) as the primary measuring device. A 5 micro-meter hydrogen-containing electrode is inserted into the H-CM for H_2_ concentration measurement.

### Cell culture

Ishikawa cell line was cultured in our lab of Shanghai General Hospital affiliated to Shanghai Jiao Tong University since Oct, 2017, catalogue number: GCC-UT0004RT/CS. HEC1A cell line was purchased from Cell Bank of the Chinese Academy of Sciences in January, 2016, catalogue number: TCHu149. AN3CA cell line was purchased from ATCC in Mar, 2016, catalogue number: ATCC®HTB-111. All the above cell lines have recently been tested for no mycoplasma contamination, and were authenticated using Short Tandem Repeat (STR) analysis as described in 2012 in ANSI Standard (ASN-0002) by the ATCC Standards Development Organization (SDO) and in Capes-Davis’s reference [31], according to the GENECHEM performs STR Profiling following ISO 9001:2008 and ISO/IEC 17025:2005 quality standards by GeneMapper Software 5. All the cell lines have ethics approval for their use by the Ethical Committee on Human Research of Shanghai General Hospital affiliated to Shanghai Jiao Tong University. Cells were cultured in DMEM for 24 h or 48 h: F12, containing 10% FBS, 100 U/mL of penicillin G, and 100 μg/mL of streptomycin at 37 °C in a humidified atmosphere of 5% CO_2_.

Secretory phase endometrium specimens were taken from 3 cases of patients who underwent curettage due to abnormal uterine bleeding in Shanghai General hospital affiliated to Shanghai Jiao Tong University in October 2018. For primary human endometrial glandular cells culture, 3 to 5 g endometrial tissue was scraped and placed in DMEM: F12, double antibiotics (penicillin 100 U/ml + streptomycin 100 U/m1) and metronidazole in a sterile collection tube. Then tissue was washed 3 times with D-Hank’s solution, double antibiotics, 100 U/ml metronidazole, and soaked for 5 min before cell culture solution was added to remove red blood cells under super clean operation; Next, the tissue was cut into 2~3 mm^3^ pieces, digested with 0.1% trypsin-EDTA, for 30 min at 37 °C, shaken every 10 min; Filter solution and 400 mesh (38 pupils) was added to remove mucus, undigested tissue, epithelial and interstitial cells. Centrifuged at 1000 rpm for 10 min, discarded the supernatant. Finally, the endometrial gland cells were resuspended and cultured into DMEM: F12 containing 10% FBS, placed at 37 °C incubator with a relative volume fraction of 5% CO_2_ for use.

Both endometrial cancer and primary human endometrial glandular cell lines were cultured with fresh H-CM for 24 h or 48 h to ensure that the cells are in good condition. About 20 min before each the follow-up functional experiment, we changed fresh H-CM again to ensure the duration of H_2_ in the medium. Hydrogen concentration detector was used for H_2_ measurement in the medium at the beginning of each functional experiment.

### Immunohistochemical staining

We retrieved endometrial cancer (*n* = 16), atypical hyperplasia (*n* = 3), and benign secretory phase endometrial tissue (*n* = 6) from hysterectomy or curettage in Shanghai General Hospital affiliated to Shanghai Jiao Tong University from May to November in 2018, and our study methodologies were approved by the Ethical Committee on Human Research of Shanghai General Hospital affiliated to Shanghai Jiao Tong University. We observed the privacy rights of human subjects. All the participants in the study confirmed and written the consent about the forms of personally identifiable data including biomedical, clinical, and biometric data. The experiments were in compliance with the Helsinki Declaration (https://www.wma.net/policies-post/wma-declaration-of-helsinki-ethical-principles-for-medical-research-involving-human-subjects/). Xenograft tumor tissue was excised from mice sacrificed by cervical dislocation, endometrial specimens were randomly examined by two independent investigators. For immunohistochemical (IHC) analysis, tissues were formalin-fixed and paraffin- embedded. Antigen was retrieved in citrate buffer (pH 6.0) after boiling, incubated with 0.01% Triton-X100 for 30 min, and 5% bovine serum albumin for 20 min. Anti-rabbit NLRP3 (1:200), anti-human caspase-1 (1:200), anti-human GSDMD (1:100), or anti-human IL-1β (1:200) primary antibodies were added overnight at 4 °C in a humidified chamber, co-incubated with biotinylated secondary antibody for 50-min. For quantitative analysis of immunohistochemistry, plaque images were visualized and analyzed with a microscopic imaging analysis system (IX71, Olympus Ltd., Japan). The immunohistochemical staining was scored for both positive cells’ proportion (0 score: 0%, 1 score: = < 10%, 2 score: 11–50%, 3 score: 51–80%, and 4 score: ≥ 80%) and staining intensity (0 score: negative, 1 score: weak, 2 score: moderate, and 3 score: strong), which ultimately resulted in designations of complete loss of expression, or weak, moderate, or strong expression, respectively.

### Western immunoblotting analysis

Total protein was lysed and extracted using RIPA buffer. Cellular protein was separated on SDS-polyacrylamide mini-gels and transferred onto a PVDF membrane for electrophoresis for 1.5 h at 300 mA. The primary antibodies used included anti-rabbit NLRP3 (1:1000), anti-mouse ASC (1:1000), anti-human caspase-1 (1:1000), anti-human GSDMD (1:1000), and anti-human IL-1β (1:1000) for 30 min. Block membranes with 5% BSA for 1 h, add primary antibody overnight at 4 °C. Secondary antibody (1:5000) was incubated with the membranes for 1 h at room temperature, followed by washing with Tris-buffered saline (TBST) three times. ECL chemiluminescence and ImageJ software was used for visualizion and quantification.

### MTT assay

Cell viability was analyzed by MTT assay. Twenty-four hrs after transfection, endometrial cancer cells were incubated with 20 μl of 5 mg/ml MTT (3–4,5-dimethylthiazol2-yl-2,5- diphenyltetrazolium bromide) reagents, 10% FBS in a 96-well plates at nearly 2000 cells per well at 37 °C for 4 h. After removing the medium from each well, the formazan granulars in the wells were dissolved with 100 μl dimethyl suiphoxide (DMSO), absorbance was measured at 490 nm spectrophotometrically. Each experiment was performed in triplicate.

### Measurements of intracellular ROS levels

Total intracellular reactive oxygen species (ROS) was determined by staining cells with the dichlorofluorescin diacetate (DCFH-DA) method [32]. Cells were incubated with 10 μM DCFH-DA for 20 min at 37 °C, mixed with 100 μL cell lysis buffer thoroughly. Each 150 μL lysate mixtures were incubated at room temperature for 5 min and transferred to a separated 96-well plate for measurement using a fluorescence microscope at 485 and 500 nm excitation and emission wavelengths.

### Measurements of mitochondrial ROS levels

Mitochondrial ROS was measured with the MitoSOX™ Red assay method. First, 50 μg MitoSOX™ mitochondrial superoxide indicator was dissolved in 5 mM MitoSOX™ reagent stock solution, incubated with 10 uM MitoSOX™ reagent at 37 °C for 10 min. Stained cells were counterstained with Tecan M100 and in warm buffer for fluorescence imaging.

### Propidium iodide (PI) staining

Cells were cultured in 12-well plates, washed twice with cold PBS, levitated in 500 μl binding buffer. Add 5 ng/mL Propidium iodide (PI) for detecting cell membrane integrity, protect it from light for 15 min at room temperature. Cells were stained with PI and analyzed by flow cytometer, immediately. Static bright-field photos of pyroptotic cells were captured and processed by Olympus IX71 and ImageJ software. Positive cells/total cells × 100% represented the PI positive cell proportion, in contrast to the untreated cells with PI staining on background.

### TUNEL assay

TUNEL-positive nuclei was identified as nicks in DNA from stained cells using the colorimetric TdT-mediated dUTP Nick-End Labeling (TUNEL) Apoptosis Assay Kit to label the terminal end of nucleic acids according to the manufacturer instructions. TUNEL-positive cells in every 100 cells was counted in three randomly selected fields per vision via microscopy.

### Measurement of lactate dehydrogenase (LDH) release

Total amount of intracellular LDH proportion under non-stimulated conditions was regarded as extracellular LDH activity. The extracellular lactate dehydrogenase (LDH) was measured to detect the pyroptotic cell death by LDH Cytotoxicity Assay Kit according to the manufacturer’s instructions. Cells were cultured at a density of 6 × 10^3^ cells/well in H-CM and CM and incubated for 12 h, 1 μg/mL doxycycline was added, supernatants and cell lysates were collected to analyze LDH release after 48 h.

### IL-1β enzyme-linked immunosorbent assay (ELISA)

HEC1A, AN3CA, and Ishikawa cells lysate or culture medium was analyzed for IL-1β using a Human Elisa Assay Kits for IL-1β according to the manufacturer’s instructions. Cells were cultured in 24-well plates overnight, 100 ng/mL of LPS was used to activate cells at 4 °C for 4 h to stimulate NLRP3 inflammasome, primed with 10 μM of nigericin for 2 h. In order to generate cell-free medium to extricate death cell, we collect the cell supernatants, centrifuge it at 300 g, 4 °C for 8 min, concentrate the supernatants, repeatedly centrifuge it at 12,000 g, 10 kDa cut-off, 4 °C for 30 min. IL-1β levels were measured at 450 nm from R&D Systems.

### GSDMD shRNA

We designed three short hairpin RNA (shRNA) sequences and detected the shRNA expression cassettes according to the small interfering (siRNA) sequences by Shanghai GeneChem Co., Ltd. Annealed oligo sequences were inserted into the digested GV493 vectors between the Phu6 and Pubi sites to generate the shRNA vectors. The lentiviral vector GV493 or GSDMD-specific shRNA particles in 6-well plates of 6 mg/mL of polybrene using a GV493 plasmid (2 μl at 1.5 × 10^9^ TU/mL, 3.75 μL at 8 × 10^8^ TU/mL, and 5 μL at 6 × 10^8^ TU/mL) was used to infect Ishikawa cells, 2 mg/mL of puromycin was added to generate stable clones, compared with an empty vector. Reverse transcription-quantitative polymerase chain reaction (RT-qPCR) was applied to confirm GSDMD gene (NM_024736) expression and protein levels.

### RT-qPCR

We selected GSDMD mRNA primers and tested them at different concentrations. Endometrial cancer cells were used to extract total RNA with a reverse transcription kit by the TRIzol method using SYBR-Green and fluorescence microscopy. The GSDMD RNA was reversed into cDNA through thermocycling at 42 °C for 1 h: denaturated at 95 °C and annealed at 60 °C for 30 s, then primer template was extensed for 40 cycles at 72 °C for 1 min. The 2^-ΔΔcq^ method was used to calculate the relative gene expression levels. The GSDMD siRNA sequences (forward primer, 5′- ACG GGC AGA GGT GGA GAC CAT − 3′; and reverse, 5′- ATG GTC TCC ACC TCT GCC CGT − 3′) was normalized to the expression of GAPDH (forward primer, 5′- TGA CTT CAA CAG CGA CAC CCA − 3′; and reverse, 5′- CAC CCT GTT GCT GTA GCC AAA-3′).

### In vivo tumorigenesis

The 4 weeks age female SPF grade BALB/c-nude mice were purchased from Shanghai Ling Chang BioTech Co., Ltd. affiliated to Shanghai Slake Laboratory Animal Co., Ltd. from Mar, 2019, License number: SCXK (shanghai) 2018–0003 and used as a human tumor xenograft model; The nude mice are homozygous mutations, hairless and thymic defects which belong to T-lymphocyte dysfunction animals. The protocols were approved by the Ethical Committee on Human Research of Shanghai General Hospital affiliated to Shanghai Jiao Tong University, China, reference number: 2019SQ054. Our research was in compliance with the Helsinki Declaration and the institutional guidelines for care and use of animals. BALB/c mice weighing 18–25 g were implanted with 1 × 10^7^ AN3CA-LUC cells at right shoulder, housing under barrier environment, with each mouse in an independent ventilation cage, at 22–26 °C and humidity 45–65%, on a 12/12-h light/dark cycle (lights on at 08:00 h). Mat and feed were changed every 2–3 days. The mice were divided into two groups: 6 mice in hydrogen-rich water group with the concentration of 1.0 ppm (HRW:H1, H2, H3, H4, H5, H6), and 3 mice in purified normal control group (NC:P1, P2, P3) randomly when the tumors grew 3 to 4 mm in diameter and were visible. The mice were separately treated by gavage with either hydrogen-rich water or pure water control (20 mL/kg/d). We changed the hydrogen-rich water every 2 h each day, and the treatments were maintained for 24 days. Mouse weight and tumor volume were measured and recorded every 3–4 days. Photographs were taken on days 12, 13, and 14 of the experiment with a living imaging system.

We anesthetized and sacrificed the animals with an overdose of 2% sodium pentobarbital (0.5 mL), and then used cervical dislocation to confirm death. We took the endometrial cancer tissues from mice from different groups and performed immunohistochemistry to determine NLRP3, caspase-1, GSDMD, and IL-1β protein expression as described previously. All samples were histologically diagnosed as endometrial cancer.

### Statistical analysis

Chi-square test is conducted to analyze the differences in IHC between groups. Differences in the patterns among different groups in Western Blot, MTT assay, ROS, mt ROS, PI, TUNEL, RT-PCR, LDH, ELISA, in vivo tumorigenesis were assessed using *t* test, multiple t test and two-way ANOVAs with multiple-comparison via Tukey’s post hoc test. Statistically significant was considered as *P* < 0.05.

### Materials

DMEM: F12 (1:1, Gibco, USA),

FBS: fetal bovine serum (Gibco, Gaithersburg, MD, USA),

streptomycin (Life Technologies, Inc., Rockville, MD),

Triton-X100(Weiao, WF0193, Shanghai, China),

anti-rabbit NLRP3 (ab210491, Abcam, USA,1:200),

anti-human caspase-1 (2225, CST, USA,1:200),

anti-human GSDMD (PA5–60727, ThermoFisher, USA, 1:100),

ECL chemiluminescence (Millipore).

ImageJ software (National Institutes of Health, Bethesda, MD, USA).

anti-human IL-1β (12,242, CST, USA,1:200).

PVDF membrane (Millipore, Billerica, MA, USA).

anti-mouse ASC (sc-514,414, SANTA CRUZ, USA, 1:1000),

BSA (Roche, Mannheim, Germany),

Tris-buffered saline (TBST),

secondary antibody (DAKO, K5007, Proteintech, Chicago, USA, 1:5000 dilution),

dichlorofluorescin diacetate (DCFH-DA, Beyotime, CA1410, Jiangsu, China),

MitoSOX™ reagent stock solution (iYEASEN, 40778ES50, Shanghai, China),

MTT (3–4,5-dimethylthiazol2-yl-2,5- diphenyltetrazolium bromide, Genview, USA).

fluorescence microscope (IX71, Olympus, Japan).

TUNEL Apoptosis Assay Kit (Beyotime, C1091, Jiangsu, China),

LDH Cytotoxicity Assay Kit (Beyotime, C0016, Jiangsu, China),

IL-1β ELISA kit (CSB-E08053h, Cusabio, USA),

GV493 vectors (Shanghai GeneChem Co., Ltd.),

GSDMD-specific shRNA (Shanghai GeneChem Co., Ltd.),

polybrene (Genomeditech Co., Ltd., Shanghai, China),

puromycin (Clontech Laboratories, Inc., Mountainview, CA, USA),

TRIzol (Thermo Fisher Scientific, Inc.) method,

A reverse transcription kit (Promega Corporation, Madision, WI, USA),

SYBR-Green (DRR041B; Takara Biotechnology Co., Ltd., Dalian, China),

fluorescence microscopy (model no. IX71; Olympus Corporation, Tokyo, Japan),

a living imaging system (Perkin Elmer, Lumina LT).

## Results

### Pyroptosis-related protein NLRP3, caspase-1, and GSDMD are overexpressed in endometrial cancer tissue

**Fig. 2:**
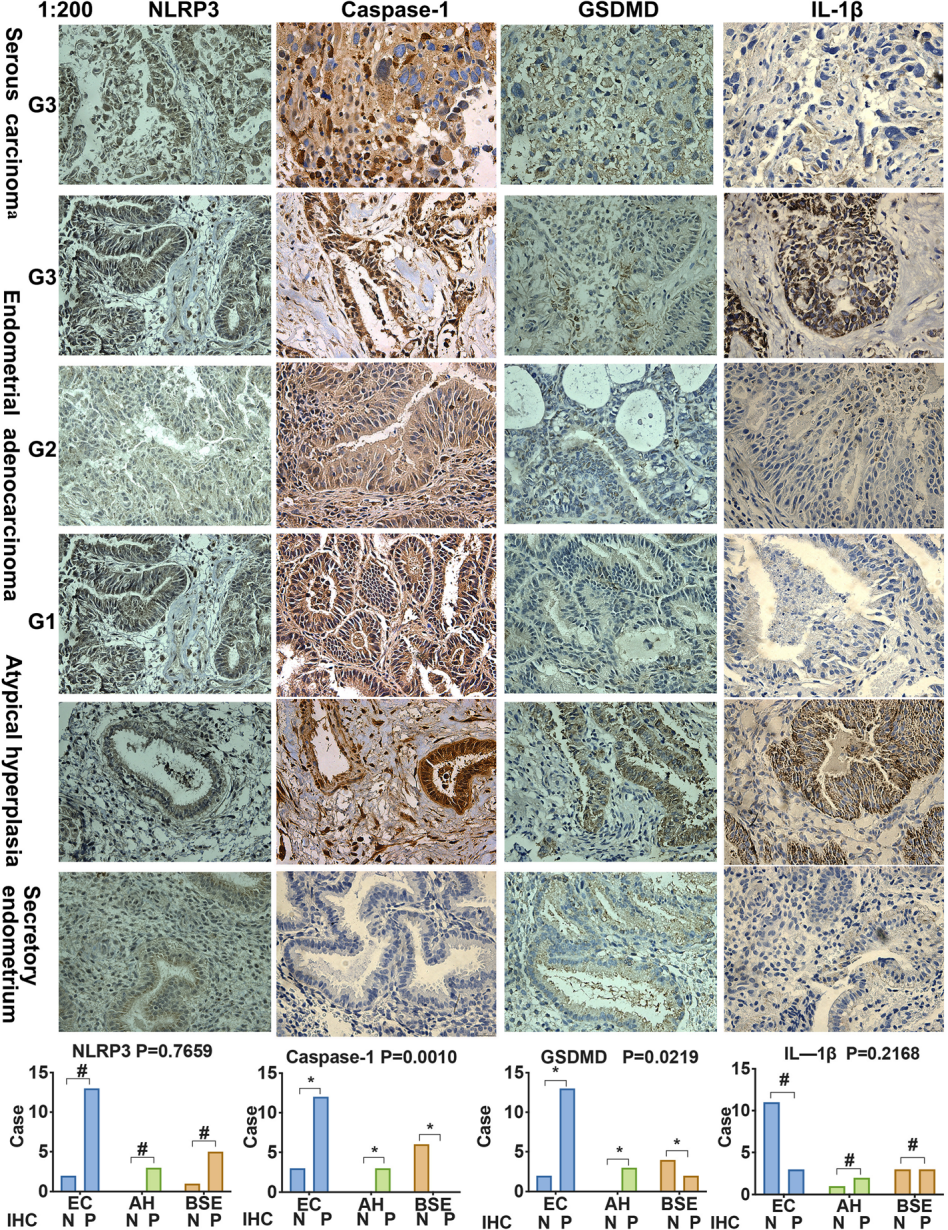
NLRP3, caspase-1, GSDMD and IL-1β are highly expressed in endometrial cancer tissues using IHC. A total of 15 endometrioid carcinoma tissues (low-grade G1, middle-grade G2, and high-grade G3), three atypical hyperplasia endometrial tissues, and six benign endometrial tissues are depicted. Cytoplasmic immunostaining of NLRP3, caspase-1, GSDMD and IL-1β in endometrial cancer tissues and secretory endometrium (magnification, 200×). Moderate -to-strong expression of NLRP3, caspase-1, and GSDMD were observed in endometrial carcinomas and atypical hyperplastic endometrial tissues, while negative or weak expression of these pyroptotic proteins were observed in benign endometrial tissues. N: negative or weak expression; P: moderate -to-strong expression; Significantly difference was observed in key pyroptotic protein caspase-1 and GSDMD. * *P* < 0.05, # *P* > 0.05 compared with the control group (See Additional file 4)

Upregulated expression of NLRP3, caspase-1, and GSDMD were detected in endometrial cancer tissues (*N* = 546) compared with normal endometrium (*N* = 35) in TCGA database, significantly difference was seen in NLRP3 and GSDMD in sample type and histological types (*P* < 0.05). There was also a trend of difference in NLRP3, caspase-1, and GSDMD protein between high and low/medium expression group in endometrial cancer related to survival rate, but there were different statistical differences between the research institutes (http://ualcan.path.uab.edu/analysis.html) (Additional file 3) [33, 34]. Based on the TCGA data above, in order to make a foundation for the follow-up hydrogen related mechanical pyroptotic experiment, we aimed to explore whether pyroptotic phenomena exist in endometrial cancer. We stained human endometrial tissue sections selected among the patients in our hospital for NLRP3, caspase-1, GSDMD, and IL-1β by IHC to examine their expression in Grade 1 (8/15), Grade 2 (3/15), and Grade 3 (4/15) endometrial cancer, atypical hyperplasia (*n* = 3), and benign endometrial tissues (*n* = 6). Two of 15 cases (13.33%) exhibited negative or weak NLRP3 staining, whereas 86.67% (13/15) of tumors manifested much higher NLRP3 staining. Negative or weak expression of caspase-1 was observed in 20% (3/15) of endometrial carcinoma cases, and 80% (12/15) showed moderate -to-strong expression, while all benign endometrial specimens showed negative or weak expression of caspase-1 (*n* = 6). Negative or weak expression of GSDMD was observed in 13.33% (2/15) of endometrial carcinoma cases, and 86.67% (13/15) showed moderate-to-strong expression. Among the 15 endometrial specimens, 73.33% (11/15) showed weak expression of IL-1β, and 20% (3/15) showed moderate expression of IL-1β. Collectively, the expression of NLRP3, caspase-1, GSDMD, and IL-1β protein was higher in cancer and atypical hyperplasia tissues than in benign endometrial tissues, significantly difference were observed in key pyroptotic protein caspase-1 and GSDMD (*P* < 0.05) (Table 1, Fig. 2). These observations indicated that a pyroptotic phenomenon does exist in endometrial cancer (Additional file 4). However, our clinical samples size was limited, further samples should be investigated to draw clinical conclusions. Additionally, we also conducted expression of pyroptotic protein in tumor tissue sections in xenograft mice model by IHC in the below experiment.

**Table 1:** Expression of NLRP3, Caspase-1 and GSDMD in endometrial tissue by IHC

	Expression/Cases	Endometrial cancer	Atypical hyperplasia	Benign endometrium
NLRP3	Negative/weak positive	2	0	4
	Medium positive	3	1	1
	Strong positive	10	2	1
Caspase-1	Negative/weak positive	3	0	6
	Medium positive	5	1	0
	Strong positive	7	2	0
GSDMD	Negative/weak positive	2	1	4
	Medium positive	5	1	1
	Strong positive	8	1	1
IL-1β	Negative/weak positive	11	1	3
	Medium positive	2	1	1
	Strong positive	1	1	2

### The NLRP3 inflammasome and GSDMD are overexpressed in hydrogen-treated endometrial cancer cells

**Fig. 3:**
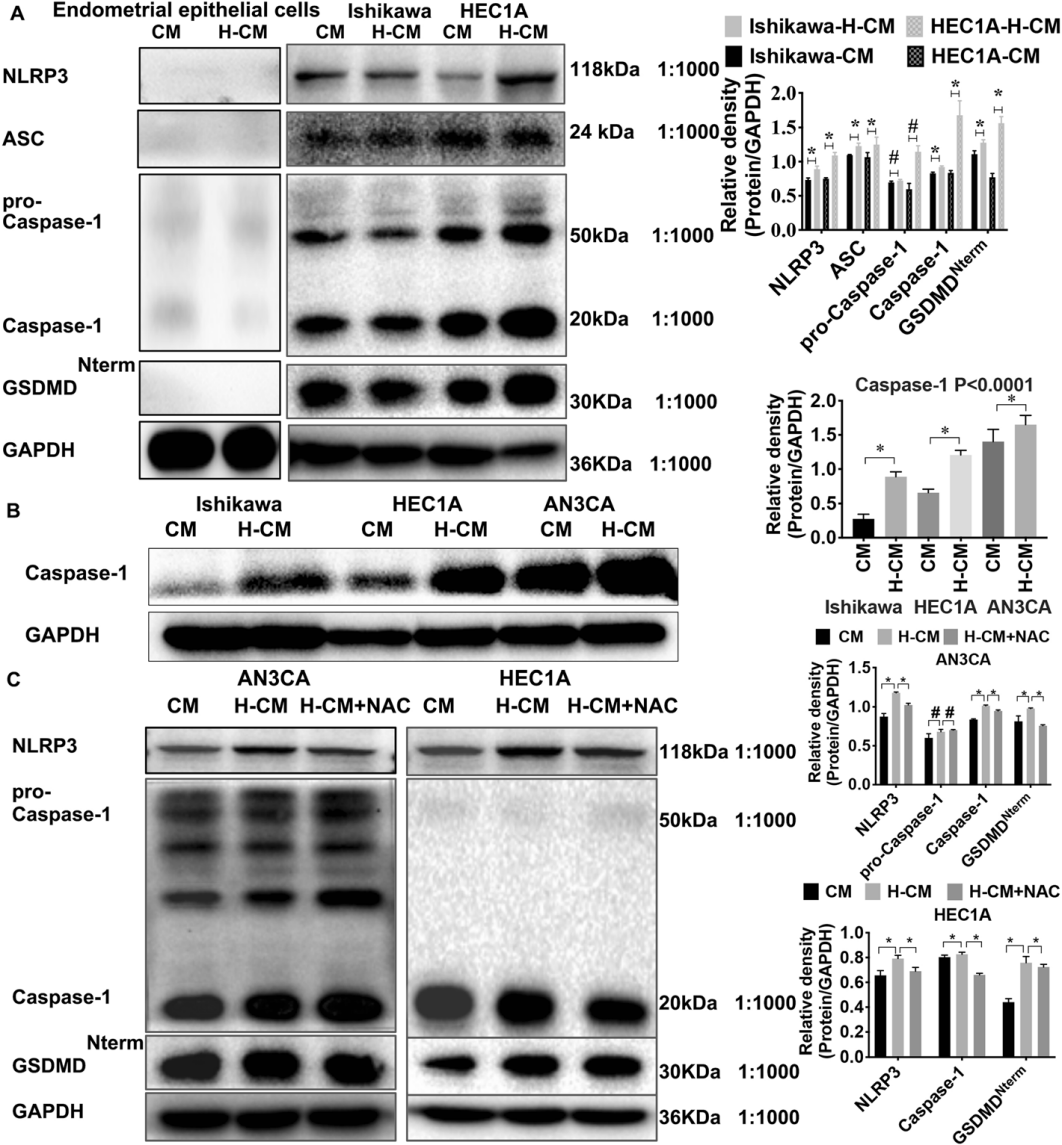
The NLRP3 inflammasome and GSDMD are increased in endometrial cancer cells. **a** Western blotting analysis revealed that levels of NLRP3, ASC, pro-caspase-1, caspase-1, and GSDMD^Nterm^ protein in Ishikawa and HEC1A cells were over-expressed after treatment with hydrogen-supplemented culture medium (H-CM) after 24 h compared with the levels in normal culture medium (CM) or in endometrial epithelial cells. **b** Expression of caspase-1 in Ishikawa, HEC1A, and AN3CA cells was significantly increased in the H-CM group compared with the level in the CM group. **c** Expression of NLRP3, pro-caspase-1, caspase-1, and GSDMD^Nterm^ was increased in hydrogen-treated AN3CA and HEC1A cells and decreased in the NAC treated H-CM group. Relative optical densities are shown beside each group. Data are expressed as mean ± S. **d** * *P* < 0.05, # *P* > 0.05 compared with the control group. Each experiment was performed in triplicate (See Additional file 5)

We observed over-expression of NLRP3, ASC, pro-caspase-1, caspase-1, and GSDMD in Ishikawa and HEC1A endometrial cancer cell lines in contrast to endometrial epithelial cells (Fig. 3a). We also investigated whether molecular hydrogen activated pyroptosis-related proteins. Hydrogenated culture medium (H-CM) was added to Ishikawa, HEC1A, and AN3CA endometrial cancer cell lines, and we identified significant statistical difference in NLRP3, ASC, caspase-1 and GSDMD^Nterm^ on relative density histogram between H-CM and normal culture medium (CM) group of ishikawa and HEC1A cells as detected by representative western blotting analyses (*P* < 0.05) (Fig. 3a).

The expression of these proteins was decreased when hydrogen-treated cells were primed with the ROS-generation blocker N-acetylcysteine (NAC, 5 mM), significant statistical difference was displayed in NLRP3, ASC, caspase-1 and GSDMD^Nterm^ on relative density histogram among CM-, H-CM-, and NAC-treated AN3CA and HEC1A cells (*P* < 0.05) (Fig. 3a-c) (Additional file 5). There was a trend in increased expression of pro-caspase-1 in hydrogen treated endometrial cancer cell lines, however significant difference was not seen (*P* > 0.05), we thought it might be related to the effect time of hydrogen on endometrial cancer cell lines which need to be further investigated. The above study indicated a possible relationship between the substantial activation effects of molecular hydrogen on the NLRP3 inflammasome and GSDMD in endometrial cancer cells, which might be related to ROS stimulation.

### Hydrogen-activated ROS generation is associated with increased expression of the NLRP3 inflammasome in endometrial cancer cells

**Fig. 4:**
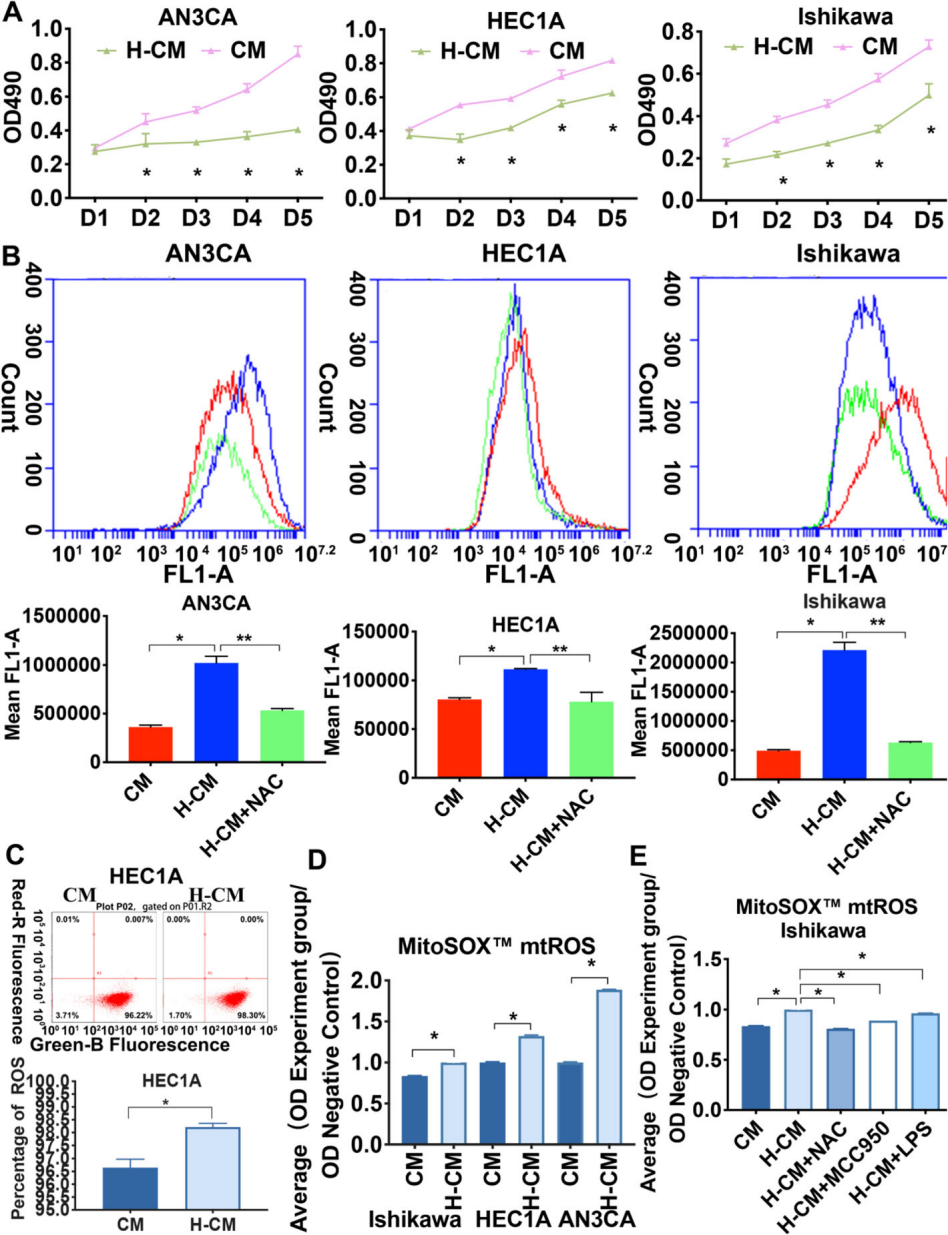
Enhanced ROS production is linked to hydrogen treatment in endometrial cancer cells. **a** Hydrogen treatment in AN3CA, HEC1A, Ishikawa cells induced decreased cell viability compared to CM cultured cells in a time-dependent manner at 48,72,96 and 120 h measured spectrophotometrically at 490 nm by MTT assay. **b** Upregulation of ROS as detected by the DCFH-DA method, AN3CA endometrial cancer cells, HEC1A cells, and AN3CA cells showed higher ROS levels upon treatment with hydrogen after 48 h, as recorded with mean FL1-A, and further decreased with NAC treatment. **c** Upregulation of ROS was observed in H-CM-treated HEC1A cells compared with the CM group. **d** Generation of mtROS was upregulated in H-CM-treated Ishikawa, HEC1A, and AN3CA cells compared with the CM group as measured with the MitoSOX™Red assay method and index ratios. **e** Ishikawa cells treated with MCC950 and NAC showed decreased expression of mtROS, while mtROS levels were upregulated in the LPS-primed group compared with the H-CM group. Data are expressed as mean ± S.D. NAC (5 mM) blocked the generation of ROS. The NLRP3 inhibitor MCC950 (10 nM) is known to potently inhibit NLRP3, and LPS (100 ng/mL) can stimulate NLRP3. Data are expressed as mean ± S.D. *, ** *P* < 0.05 compared with the control group. Each experiment was performed in triplicate (See Additional file 6)

First, in order to analysis cell viability in hydrogen-treated endometrial cancer cells after transfection (24, 48, 72, 96 and 120 h), MTT assay was used. Each solution was measured spectrophotometrically at 490 nm. Hydrogen treatment in AN3CA, HEC1A, Ishikawa cells induced decreased cell viability compared to CM cultured cells in a time-dependent manner at 48,72,96 and 120 h (*P* < 0.05) (Fig. 4a, Additional file 6).

Since the initial pyroptotic signals ROS and the NLRP3 inflammasome were potential targets of molecular hydrogen, and because increased tumor cell ROS could promote cancer cell death, we investigated whether treatment with hydrogen was able to boost ROS accumulation in endometrial cancer cells. We observed that Ishikawa, HEC1A, and AN3CA cells treated with H-CM displayed significantly increased generation of ROS after 20 min compared with the CM group using the DCFH-DA method (*P* < 0.05). In contrast, NAC decreased ROS levels in hydrogen-treated endometrial cancer cells (*P* < 0.05) (Fig. 4b, c). The mitochondrion is the major organelle that generates ROS, and we observed an increased generation of mitochondrial (mt) ROS levels in AN3CA, HEC1A, and Ishikawa cells compared with the control group as measured with the MitoSOX™Red assay method (*P* < 0.05) (Fig. 4d). We therefore concluded that molecular hydrogen activated ROS and mtROS generation in endometrial cancer cells.

We next evaluated whether the activation effect of molecular hydrogen on NLRP3 arises from its capacity to stimulate mtROS, using the potent NLRP3 inhibitor MCC950 and LPS, which is an NLRP3 activator. Pretreatment with NAC and MCC950 reduced the hydrogen-induced production of mtROS in Ishikawa cells (*P* < 0.05), while mtROS levels were upregulated in LPS-treated cells (*P* < 0.05) (Fig. 4e) (Additional file 6). These results indicated that NLRP3 inflammasome activation was tightly associated with its mtROS activity under hydrogen stimulation.

### Hydrogen-induced ROS and NLRP3 inflammasome-mediated pyroptosis in endometrial cancer

**Fig. 5:**
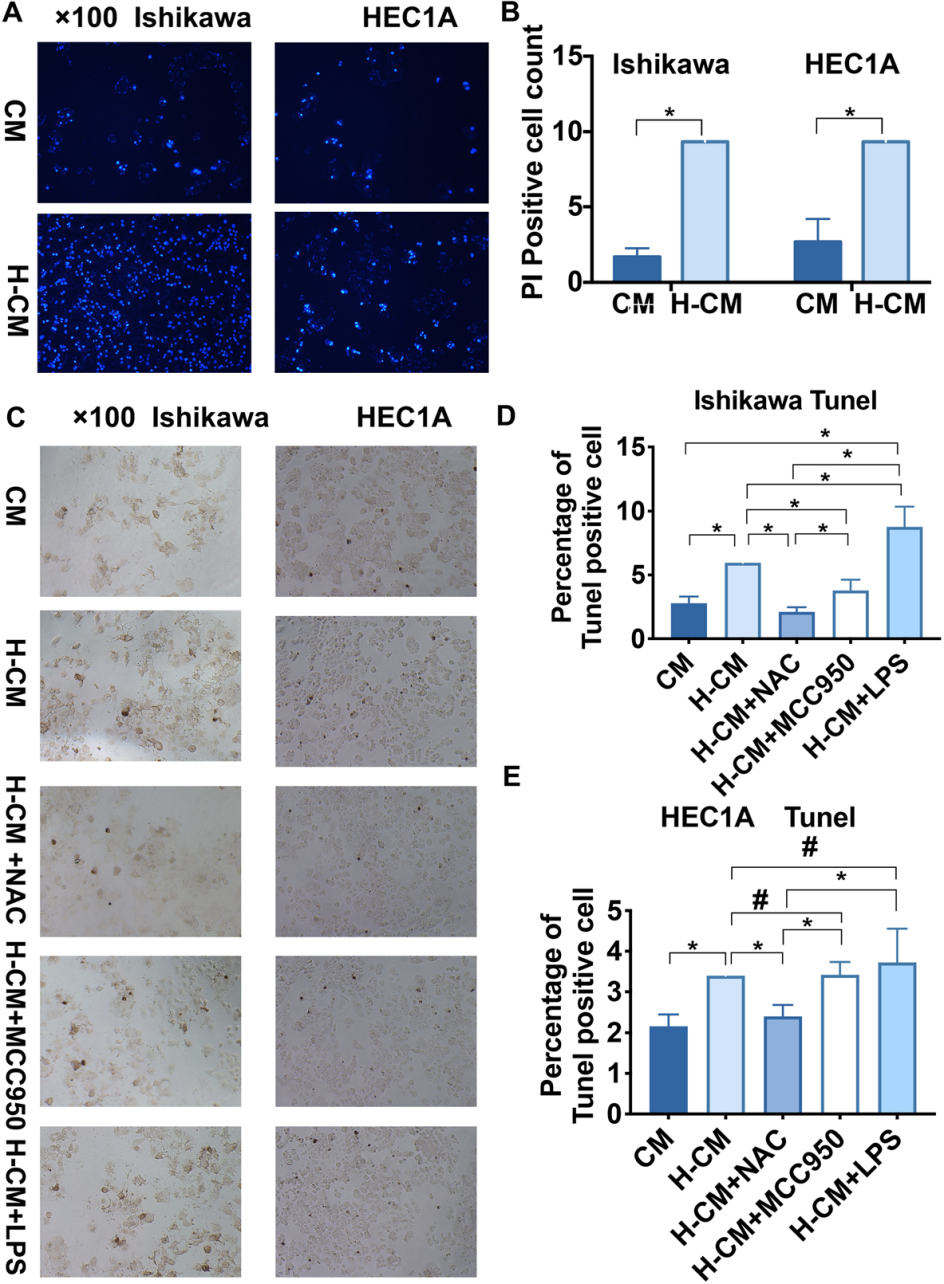
Hydrogen-activated endometrial cancer pyroptosis via ROS and the NLRP3 inflammasome. Pyroptotic cell death was demonstrated by the percentage of PI- and TUNEL-positive cells. **a** and **b** Hydrogen pretreatment for 24 h increased the number of PI-staining Ishikawa and HEC1A cells. **c**, **d**, **e** The percentage of TUNEL-positive cells was increased in H-CM-treated Ishikawa (**d**) and HEC1A cells (**e**) compared with the CM-treated group. H-CM-treated Ishikawa (**d**) and HEC1A cells (**e**) primed with NAC (5 nM) decreased the percentage of TUNEL-positive cells. Compared with H-CM group, MCC950 (10 nM) treatment of H-CM Ishikawa (**d**) and HEC1A cells (**e**) also decreased the percentage of TUNEL-positive cells, and LPS (10 ng/mL) treatment of H-CM Ishikawa (**d**) and HEC1A cells (**e**) increased the percentage of TUNEL-positive cells within 6 h. Data are expressed as mean ± S.D. *P < 0.05, # *P* > 0.05. Each experiment was performed in triplicate (See Additional file 7)

To identify pyroptotic cell death in hydrogen-treated endometrial cancer cells, PI and TUNEL staining were conducted to detect cell membrane swelling and DNA damage, respectively. Our results showed increased and extensive PI-positive- staining cells in hydrogen-treated Ishikawa cells compared with the control group (*P* < 0.05), and similar results were observed in hydrogen-treated HEC1A cells (*P* < 0.05) (Fig. 5a, b). Next we performed a TUNEL assay to detect DNA fragmentation resulting from pyroptotic cascades. As shown in Fig. 5, we observed significantly more TUNEL-positive cells in hydrogen-treated Ishikawa and HEC1A cells (*P* < 0.05) compared with CM group. These results strongly suggested that hydrogen activated pyroptosis. TUNEL-positive cells were markedly downregulated in H-CM-Ishikawa and HEC1A cells within 6 h after treatment with 5 mM NAC (*P* < 0.05) (Fig. 5c, d), indicating that ROS activated pyroptosis. In order to further characterize the relationship between the NLRP3 inflammasome and ROS under hydrogen stimulation, we noted that MCC950 and LPS treatment of Ishikawa and HEC1A cells cultured in H-CM resulted in a decreased and increased percentage of TUNEL-positive cells within 6 h compared with NAC treatment group (*P* < 0.05), respectively. MCC950 and LPS treatment of Ishikawa cells cultured in H-CM also resulted in a significantly decreased and increased percentage of TUNEL-positive cells (*P* < 0.05) compared with CM group, similar trend were also observed in HEC1A cells, while significant difference did not show, which need further research (Fig. 5c, d, e, Additional file 7). These results implied that molecular hydrogen might induce ROS- and NLRP3 inflammasome-mediated pyroptosis in endometrial cancer.

### Identification of GSDMD as a required component for pyroptosis

**Fig. 6:**
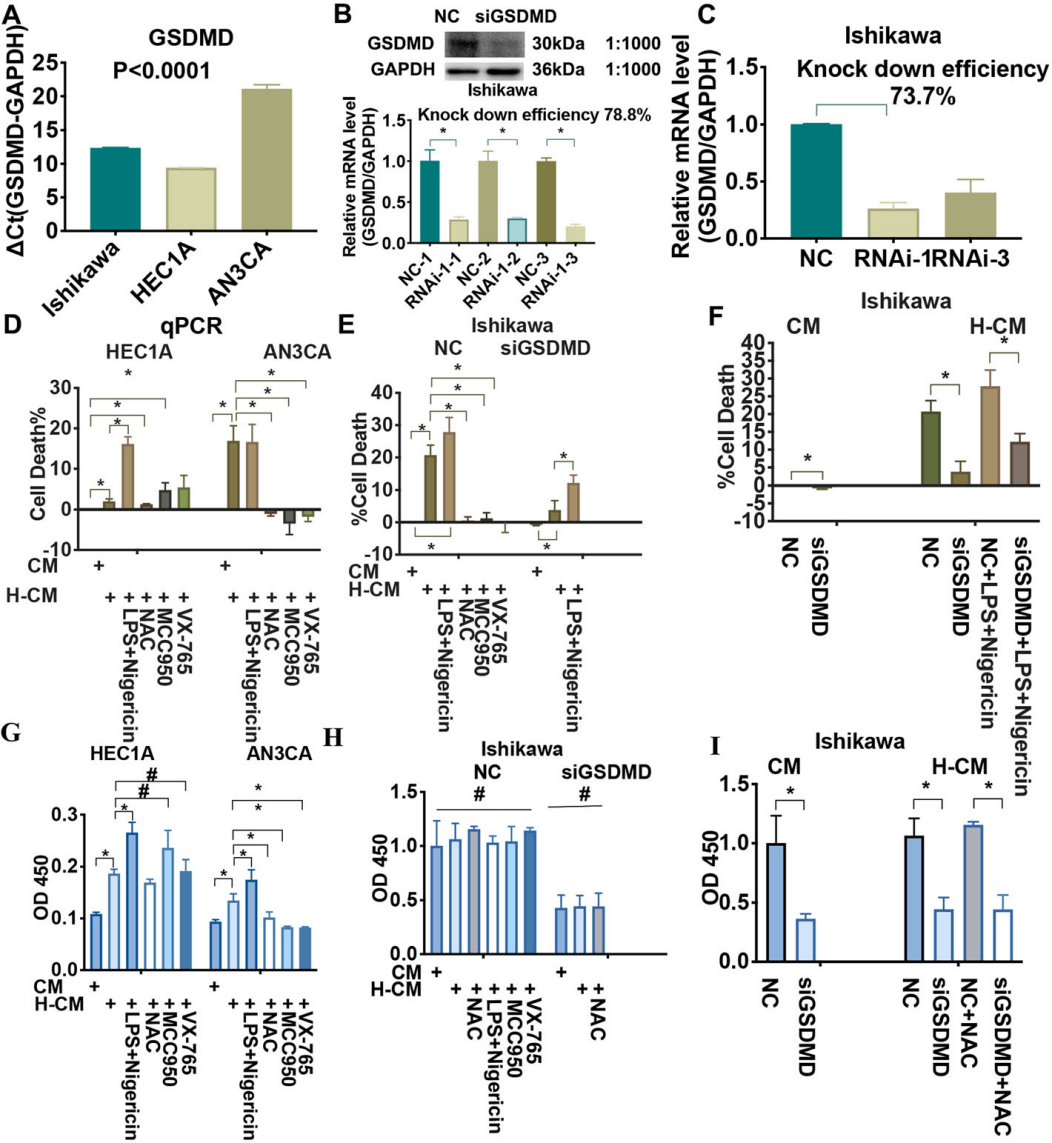
Knockdown of GSDMD by lentiviral shRNA. Hydrogen mediated GSDMD triggers pyroptosis in endometrial cancer cells. Three pairs of GSDMD gene shRNA interference fragments: LV-GSDMD-RNA interference (i)-71,359–11 (RNAi-1), LV-GSDMD-RNAi-71,360-1 (RNAi-2), and LV-GSDMD-RNAi-71,361–1 (RNAi-3), and one negative control (NC) pair were designed and transfected into Ishikawa cells. **A**. GSDMD mRNA expression in Ishikawa, HEC1A, and AN3CA cells, and **B**. GSDMD mRNA expression in Ishikawa cells following transfection with RNAi-1 or RNAi-3 were decreased significantly compared with that in the NC group. Two shRNA vectors suppressed GSDMD mRNA expression by 73.7 and 59.6%, respectively. Western blotting analysis revealed that levels of GSDMD^Nterm^ protein in GSDMD depleted ishikawa cells were downregulated compared with NC group. **C**. RNAi-1-1 had an increased transfection efficiency (78.8%) and was selected for subsequent experiments. LDH release assay (**d**, **e**, **f**) and IL-1β release assay by ELISA (**G**. **H**. **I**.) under hydrogen stimulation from HEC1A, AN3CA, and Ishikawa cells primed with LPS (10 ng/mL for 4 h), and then stimulated for 30 min with nigericin (10 μM), NAC (5 mM), MCC950 (10 μM), or VX-765 (0.8 nM). **d**. Compared with CM group, hydrogen treatment after 4 h significantly induced the release of LDH from HEC1A and AN3CA cells. LDH release increased after hydrogen-treated HEC1A cells were primed with LPS and nigericin, but decreased after AN3CA cells were primed with NAC, MCC950, or VX-765. **e**. Compared with CM group, hydrogen treatment or primed with LPS and nigericin significantly induced the release of LDH from Ishikawa cells. Compared with H-CM group, LDH release decreased after cells were primed with NAC, MCC950, or VX-765. Even if GSDMD was deleted, H-CM-Ishikawa cells primed with LPS and nigericin produced a greater release of LDH. **f**. GSDMD depletion reduced the release of LDH with both H-CM and CM treatment or with LPS and nigericin in Ishikawa cells compared with the control group. **G**. Hydrogen treatment enhanced significantly the release of IL-1β from HEC1A and AN3CA cells compared with CM group. IL-1β release increased after hydrogen treatment in HEC1A and AN3CA cells primed with LPS and nigericin, while release diminished after AN3CA cells were primed with NAC, MCC950, or VX-765 compared with H-CM group. **h**. There were, however, no significant differences on the release of IL-1β among the different groups with or without hydrogen treatment when a GSDMD siRNA was transfected into Ishikawa cells. **i**. GSDMD depletion reduced the release of IL-1β in CM-, H-CM- and NAC-treated Ishikawa cells compared with the control group. **I**. *P < 0.05, # *P* > 0.05. Each experiment was performed in triplicate (See Additional file 8, 9, and 10)

During pyroptosis, pores formed in cell membranes and cellular contents were released as dead cells staining. Therefore, we next measured the amount of lactate dehydrogenase (LDH) and IL-1β release as indices of osmotic lysis, which can be determined by the percentage of cell death and the OD450 value, respectively. We investigated whether hydrogen determined pyroptotic cell death by ROS and the NLRP3 inflammasome, or whether GSDMD^Nterm^ expression induced a terminal lytic type of cell death that is characteristic of pyroptosis. We executed GSDMD knockdown experiments by using a lentivirus that carries a GSDMD shRNA to transfect Ishikawa cells, including three pairs of GSDMD gene shRNA interference fragments (RNAi-1, RNAi-2, and RNAi-3), as well as one negative control (NC) pair. Western blotting analysis revealed that levels of GSDMD^Nterm^ protein in GSDMD depleted ishikawa cells were downregulated compared with NC group (Fig. 6b). The RNAi-1 with an increased transfection efficiency (78.8%) was selected for subsequent experiments (Fig. 6a-c, Additional file 8).

Hydrogen treatment increased the percentage of cell death (Fig. 6d) and the OD450 value (Fig. 6g) significantly compared with the normal culture group in HEC1A and AN3CA cells. In Ishikawa cells, hydrogen treatment stimulated significantly higher LDH release compared with the CM group (*P* < 0.05) (Fig. 6e), and the OD450 value was even higher (*P* > 0.05) (Fig. 6h). These results verified that molecular hydrogen induced pyroptosis in endometrial cancer. Both LPS and nigericin treatment further upregulated LDH and IL-1β release (*P* < 0.05), while LDH release decreased in NAC-, MCC950- or caspase-1 inhibitor VX-765-treated HEC1A and Ishikawa cells under hydrogen stimulation (P < 0.05) the H-CM group, confirming that hydrogen induced pyroptosis via the ROS-NLRP3- caspase-1 pathway (Fig. 6d, g).

Hydrogen treatment also upregulated LDH release in GSDMD-depleted Ishikawa cells (Fig. 6e), while failed to induce IL-1β release (Fig.6h). When GSDMD was deleted in hydrogen-treated Ishikawa cells, LPS and nigericin augmented the release of LDH (*P* < 0.05) (Fig. 6e), while NAC failed to induce IL-1β release (Fig.6h). GSDMD depletion reduced the release of LDH (Fig. 6f) and the production of IL-1β (Fig. 6i) in both H-CM- and CM-treated Ishikawa cells compared with the control group (*P* < 0.05). We observed similar results in the LPS- and nigericin- or NAC-treated Ishikawa cells, indicating that GSDMD deletion blocked pyroptotic cell death in endometrial cancer cells (*P* < 0.05). However, there was no significant difference on IL-1β release among different groups of Ishikawa cells with or without hydrogen treatment when GSDMD was silenced (Fig. 6h) (Additional file 9 and Additional file 10). Therefore, we hypothesize that GSDMD is the terminal protein in the pyroptotic sequence in endometrial cancer cells, and that GSDMD-mediated IL-1β secretion occurs via the ROS-NLRP3-caspase-1 signaling pathway is induced by activating effector molecular hydrogen.

### Hydrogen-rich water treatment inhibits endometrial tumorigenesis in vivo

We next aimed to determine whether drinking hydrogen-rich water would inhibit tumor growth in a mouse model of endometrial cancer. Nine BALB/c mice were subcutaneously implanted with 1 × 10^7^ AN3CA-LUC cells at right shoulder, and fed with either hydrogen-rich water with the concentration of 1.0 ppm (HRW:H1,H2,H3,H4,H5,H6) or purified normal control (NC:P1, P2, P3) water ad lib each day. The tumor started growing on H1, H2, H3 and P1, P2, P3 mice on 31st Mar, 2019, while the tumor started growing on H4, H5 and H6 mice on 13th May, 2019. Compliance with ethics, H1, H3 and P2 mice were sacrificed as volume increased to 770.73–901.69 mm^3^ on day 14, H4, H5 and H6 mice were sacrificed as volume increased to 2317.83–4456.70 mm^3^ on day 17, so we recorded the data of H2, P1, P3 mice for 24 days, H1, H3, P2 mice for 14 days, and H4, H5, H6 mice for 17 days .

**Fig. 7:**
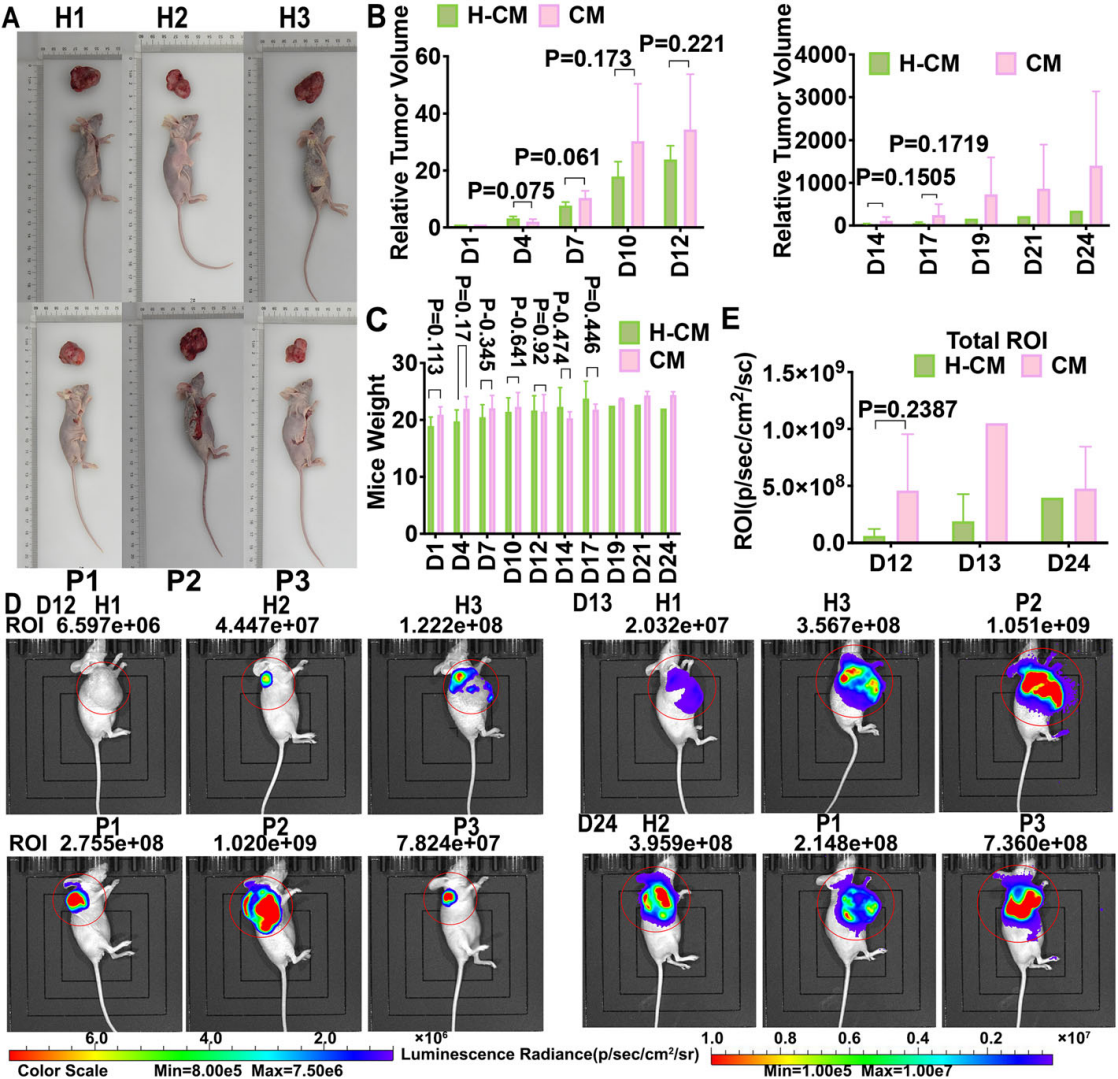
Effects of oral intake of hydrogen-rich water on xenografted mice. All nine Female BALB/c-nude mice weighing 18–25 g implanted with 1 × 10^7^ AN3CA-LUC cells at right shoulder were fed with either hydrogen-rich water (HRW) with the concentration of 1.0 ppm or control (NC) purified water (20 mL/kg/d) for 24 days. Six for HRW-fed groups (H1, H2, H3, H4, H5, H6); Three for purified water- fed groups (P1, P2, P3); **a**. The tumor volume (mm^3^) of the HRW group was decreased compared to the control group. **b**. There was a trend in the relative tumor volume in the HRW group on day 1–24 compared to the control group. **c**. The mice weight (g) in the HRW group was diminished on day 1–10, 19–24 compared to the control group. **d**. Living tumor imaging after oral intake of hydrogen-rich water in xenografted mice. Photographs show tumor imaging in two groups, each containing three AN3CA-LUC cells -implanted mice per group in Day 12 (H1, H2, H3, P1, P2, P3), Day 13 (H1, H3, P2), Day 24 (H2, P1, P3). The color scale is represented by ROI = radiance (p/sec/cm^2^/sr). The color scale changes from blue to red, with the darker the color, the greater the tumor density. **e**. Mice subjected to hydrogen-rich water (HRW) displayed a decreased ROI as assessed by luminescence analysis of Total Radiant Efficiency (Day 12: HRW vs. NC, 5.90E+ 07 vs. 4.60E+ 08). Values are mean ± SD of six mice per HRW group and three mice per NC group (See Additional file 11)

The mice weighted 18.1, 19.2, 20.1, 17.0, 21.3, 17.9 (g) in HRW group, and weighted 19.4, 22.2, 21.1(g) in NC group on day 1. Water intake was monitored throughout the experimental period and was nearly the same among groups. Hydrogen-rich water administration reduced endometrial tumor growth and tumor volume (mm^3^) (Fig. 7a). The mice weight (g) in the HRW group was diminished on most of the observed days (day 1–10, 19–24) compared to the control group (Fig. 7c). The speed of tumor volume increase time also gradually declined in HRW group on day 7, 14–19 (Additional file 11). We thought the reason why mice weight and tumor volume did not consistently decrease on all the observed days was due to individual differences. Since the absolute mice weight and tumor volume of the nine mice on day 1 was different, in order to balance individual differences, we wondered it was more meaningful to observe the relative increase in tumor volume. Thus tumor volume on the first day were defined as 1 and the durations of increased or decreased proportions were recorded as the relative tumor volume. We observed a decreased trend in the relative tumor volume in the HRW group on all the observed days (day 1–24) compared with the control group (Fig. 7b), (Additional file 11). These observations indicated the phenomenon that hydrogen supplementation in mice could inhibit xenograft volume and weight of endometrial tumors. However, no statistical difference was found in xenograft mice model study since there were limitations in the sample size, which needed to be expanded for further research. We next investigated the radiance (ROI) of the tumor of mice to verify the antitumor effect of hydrogen on endometrial cancer.

We demonstrated that drinking hydrogen-rich water reduced the volume of endometrial tumors in a xenograft mouse model. The mice that we subjected to hydrogen-rich water (HRW) displayed decreased radiance (ROI) after 12 days, as assessed by luminescence analysis, thus indicating lower tumor density (Fig. 7d). Mouse H1, H3, and P2 were sacrificed on day 13, while mouse H2 and P1 were sacrificed on day 24. Due to limitations in the sample size, we observed a decrease trend in total ROI in the HRW group on day 12, 13, 24 (Fig. 7e), (Additional file 1). Our study thus demonstrated that hydrogen plays an antitumor role in endometrial cancer. Next we conducted IHC staining to explore whether the hydrogen-induced anti-tumor effect was related to pyroptosis.

### Pyroptosis-related protein NLRP3, caspase-1, and GSDMD are overexpressed in endometrial cancer tissue of a hydrogen-rich drinking water xenograft mouse model

**Fig. 8:**
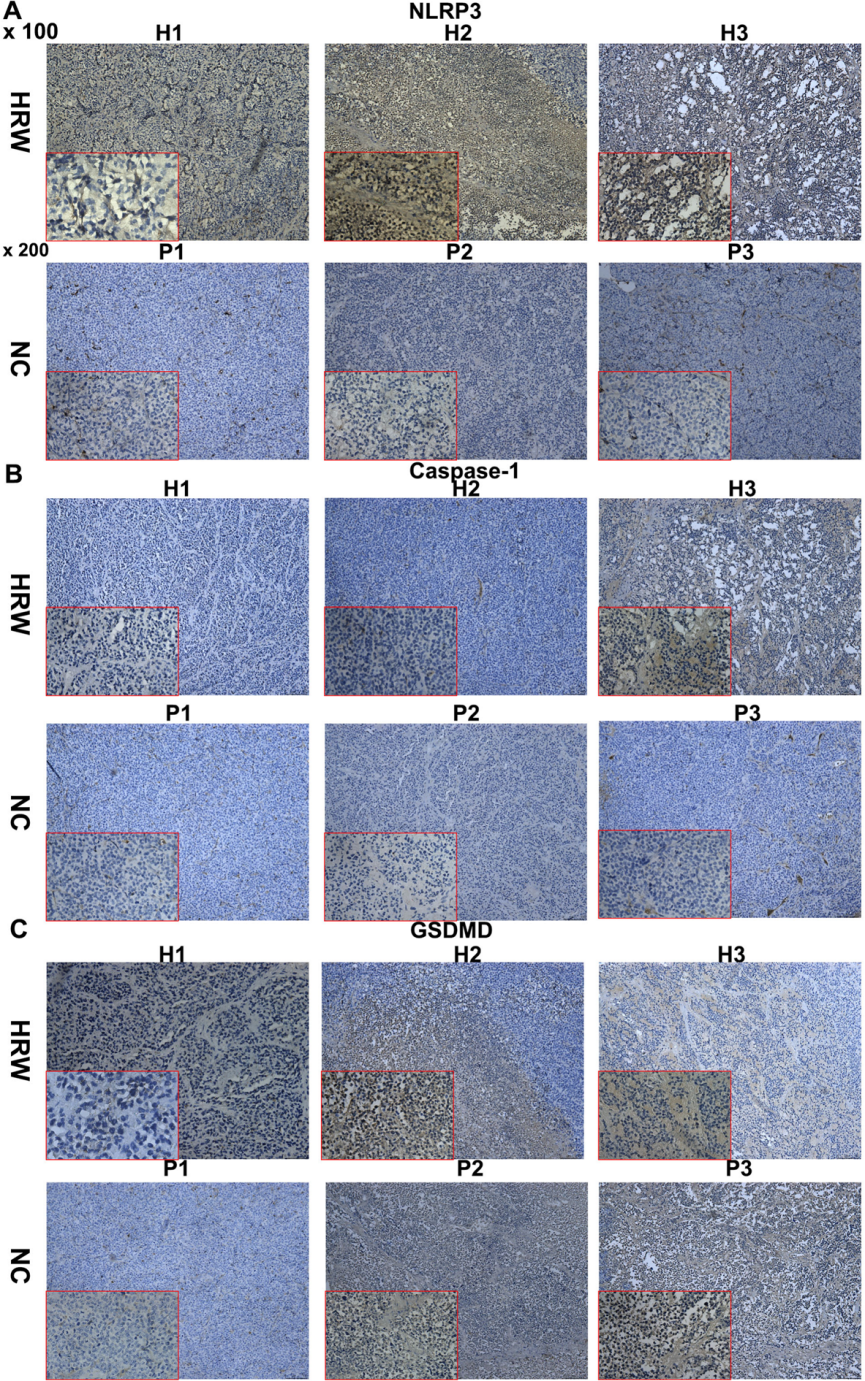
NLRP3, caspase-1 and GSDMD are highly expressed in endometrial cancer tissue of the hydrogen-rich drinking water xenograft mouse model. Tumor tissue sections in HRW and NC group presented moderate -to-strong expression of NLRP3 (**a**), caspase-1 (**b**) and GSDMD (**c**), HRW group expressed much higher staining on IHC. Images are presented at ×100 magnification and insets at ×200 magnification

Next, we explored whether the hydrogen induced pyroptotic pathway affected endometrial tumorigenesis in vivo. Though tumor tissue sections in the HRW and NC groups presented moderate-to-strong positive expression of NLRP3 (Fig. 8a), caspase-1 (Fig. 8b) and GSDMD (Fig. 8c), the HRW group showed much higher staining by IHC. These observations indicated that hydrogen attenuated tumor volume and weight in a xenograft mouse model though the pyroptotic pathway.

## Discussion

Endometrial cancers of specific pathologic types—such as serous carcinomas or cancers with high risk factors such as distant metastases or deep myometrial invasion—might be insensitive to radio/chemotherapy because of resistance to apoptosis and necrosis; thus, pyroptotic death might be another targeted therapeutic approach to endometrial cancer. According to the TCGA database, GSDMD protein expression was significantly different between endometrial cancer and normal endometrial tissue, and correlated with pathological type, stage and patient weight [33, 34]. Recent studies have shown that pyroptosis is a characteristic of autoinflammatory and autoimmune diseases [35, 36]; We found pyroptotic phenomena reflected as high expression of NLRP3, caspase-1, GSDMD, and IL-1β in endometrial cancer lesions as identified by IHC analyses.

Apoptosis, necroptosis, and pyroptosis are distinct cell death processes in host cells [18, 37]. Apoptosis is a caspase-3/7/8/9-mediated, gene-regulated programmed cell death, whereas necroptosis is a pro-inflammatory and non-caspase-mediated cell death process [38]. Pyroptosis, in contrast, is mediated by the inflammasome and caspase-1/4/11, which are found in macrophages, dendritic and neutrophils immune cells, and play critical roles in the clearance of intracellular bacteria [39] (Table 2). There are several other differences between pyroptosis and apoptosis. First, the nuclear morphology of pyroptotic cells causes DNA damage [5], but DNA laddering is not necessarily observed [40, 41]. In addition, the TUNEL assay demonstrates positivity during pyroptosis [40, 42]. Second, pyroptotic cells stain positive for annexin V, which binds to phosphatidyl serine that is normally restricted to the inner side of the cell membrane. As a result, PI-staining-pyroptotic cells can be detected [40]. Third, the additional outcome of spherical membrane pore formation in pyroptosis is cellular swelling. The cytosolic contents combine, rupture and release to the extracellular space after continued rapid swelling of these protrusions, which can be commonly measured by assaying for LDH [40]. Pyroptotic and apoptotic pathways are also linked via a direct interaction between the inflammatory and apoptotic caspases, and two cleavage sites in the GSDMD protein are currently considered checkpoints for determining infected cells progression to either pyroptosis or apoptosis [43].

**Table 2:** Comparison of apoptosis, necroptosis and pyroptosis

		Apoptosis	Necroptosis	Pyroptosis
Initiating		Program	Random	Program
Signaling pathway		Caspase-3/7/8	TNF-α	Caspase-1/11
Terminal event	Membrane pore formation	No	MLKL	GSDMD
	Cell swelling	No	Yes	Yes
	PI staining	No	Yes	Yes
	LDH release	No	Yes	Yes
	TUNEL	Yes	Yes	Yes
Tissue effect	Inflammatory	No	Yes	Yes
	IL-1β release	No	No	Yes

It is extremely important to find efficient and safe clinical applications for activating pyroptosis in endometrial cancer. Since ROS constitutes an upstream mechanism implicated in cellular pyroptosis, and because ROS concentrations are higher in endometrial cancer than in normal endometrial tissue, we hypothesize that elevated ROS will lead to activation of cell death by pyroptosis in endometrial cancer cells [44–46]. Investigators have increasingly accepted either H_2_ gas or H_2_-rich saline as promising candidates in therapeutic approaches to cancer [23]. H_2_ is a safe component of air, with a concentration of less than 4.7%, and it rapidly penetrates biologic membranes; however, H_2_ has a half-life of 0–2 h and almost disappears by 8 h [20]. Our study showed that endometrial cells stimulated with hydrogen can result in cellular and mitochondrial generation of ROS (*P* < 0.05), which has been proven to trigger NLRP3 activation [47–49].

The inflammatory response caused by the NLRP3 inflammasome is triggered in a variety of situations that pose a threat to the host, including TNF-α, the NF-κB pathway, and ROS [9, 14, 50, 51]. In our previous work, we demonstrated that hydrogen treatment activates TNF-α and the NF-κB pathway in endometrial cancer cells (Additional file 2). In the present study, we found that the NLRP3 inflammasome (including NLRP3 and caspase-1 expression) was upregulated in endometrial cancer tissue compared to that of benign tissues (Table 1, Fig. 2). Based on our findings that hydrogen treatment increased the expression of pyroptosis-related protein NLRP3, ASC, pro-caspase-1, and caspase-1 in endometrial cancer cells (Fig. 3), and that hydrogen-treated cells exhibited higher populations of PI- and TUNEL-positive cells, we propose that pyroptosis by DNA fragmentation is mediated by ROS and the NLRP3 inflammasome (Figs. 4, 5 and 6).

However, we need to further determine the key effect of hydrogen-activated pyroptosis in endometrial cancer. Clarification of this aspect will promote a deeper understanding of the detailed mechanism (s) underlying classical GSDMD-triggered pyroptosis. GSDMD is an endogenous pore-forming protein, and its actuation requires an activator. We demonstrated that H_2_ activated ROS-NLRP3-caspase-1 in the induction of GSDMD-dependent pyroptosis (Fig. 6). Accordingly, our finding triggers an interest in determining whether the activation of GSDMD by H_2_ in endometrial cancer cells ultimately triggers pyroptosis. Classical GSDMD-mediated pyroptosis includes three key steps. First, cleavage of GSDMD by caspase-1 to cut the p20 fragment C-terminal GSDMD (GSDMD^cterm^) and form the functional p30 fragment N-terminal domain (NTD) GSDMD (GSDMD^Nterm^) occurs. Second, GSDMD inserts its NTD into the plasma membrane and binds [52]. Third, a plasma membrane pore is created by GSDMD^Nterm^, with an inner diameter of 10–20 nm [18]; this is a key step in pyroptosis [52, 53]. The p30 fragment of GSDMD exists as a higher-order oligomer and forms ring-like structures which can be observed by negative-stain electron microscopy. These structures appear within minutes of GSDMD cleavage and release Ca^2+^ from preloaded liposomes [53]. After plasma membrane pores form, GSDMD triggers potassium efflux, and leads to the process of swelling, membrane rupture, release of cellular contents, nuclear chromatin condensation, DNA cleavage and fragmentation, thus releasing IL-1β and IL-18. Preventing GSDMD-induced potassium ion efflux appears to block pyroptotic cell killing [54]. We observed LDH and IL-1β release to be increased in hydrogen-treated endometrial cancer cells that were upregulated by LPS and nigericin or downregulated by ROS, NLRP3, or the caspase-1 inhibitor (Fig. 6). To elucidate the structural basis for human GSDMD action in cellular pyroptosis, we investigated N-terminal domains of the human GSDMD protein. Silencing of GSDMD resulted in decreased levels of LDH and IL-1β release, indicating that GSDMD induced death through cell lysis. When GSDMD was depleted, there was no significant difference between the hydrogen- and normal-culture-treated groups. Therefore, GSDMD might be the terminal protein that participates in pyroptosis in endometrial cancer. Our data demonstrated that GSDMD was recruited to the NLRP3 inflammasome after LPS and nigericin priming in endometrial cancer cells, and that GSDMD was required for mediation by the NLRP3 inflammasome of pyroptosis and IL-1β production (Fig. 6). We thus referred to these GSDMD-mediated membrane pores that allow hydrogen efflux as “hydrogen channels”. This latter observation suggests that there may be several underlying mechanisms by which hydrogen exerts its protective effects against endometrial cancer.

In the present study, we explored the effects of hydrogen on endometrial cancer and provided the first evidence that hydrogen induces pyroptosis via ROS-NLRP3-caspase-1 pathways. We demonstrated that drinking hydrogen-enriched water reduced the volume and weight of endometrial tumors in a xenograft mouse model. Specifically, mice subjected to hydrogen-rich water (HRW) displayed a decrease in ROI after 12 days using fluorescence quantitative analysis, indicating that imbibing hydrogen-rich water attenuated tumor density (Fig. 7). This suggests that drinking H_2_ water is effective in treating endometrial cancer. Tumor tissue sections in the HRW group presented much higher NLRP3, caspase-1 and GSDMD staining by IHC (Fig. 8).

## Conclusions

Collectively, our results suggest that hydrogen initiates GSDMD pathway-mediated pyroptosis, and that this modality could be further developed as a sensitizer to GSDMD-targeted therapy. Hence, hydrogen might exert its effect on the endometrium through both inflammation-dependent cell death mechanisms. However, there was limitations in the sample size of endometrial cancer patient and xenograft mouse model in our study. Importantly, future research should aim to analyze tumor tissue sections of the xenograft mouse model in HRW and NC group via the Tandem Mass Tag (TMT) method to explore the roles of GSDMD and hydrogen-activated pyroptosis in endometrial cancer.

## Supplementary information

**Additional file 1.** High-throughput sequencing in TCGA database. High-throughput sequencing of NF-κB pathway gene associated with GSDMD and NLRP3 in 176 endometrial cancer specimens via the TCGA database.

**Additional file 2.** Significantly affected pathways and genes. Significantly affected pathways and genes in hydrogen-treated endometrial cancer cells.

**Additional file 3.** TCGA data. NLRP3, caspase-1, and GSDMD protein expression in endometrial cancer related to survival rate based on the TCGA data.

**Additional file 4. **IHC. Expression of pyroptosis-related protein NLRP3, caspase-1, and GSDMD in endometrial cancer tissue by IHC.

**Additional file 5.** Western Blot. The expression of NLRP3 inflammasome and GSDMD in hydrogen-treated endometrial cancer cells by Western Blot.

**Additional file 6.** MTT and ROS. Cell viability (MTT) and ROS in hydrogen-treated endometrial cancer.

**Additional file 7. **PI and TUNEL assay. Hydrogen-induced ROS and NLRP3 inflammasome-mediated pyroptosis in endometrial cancer by PI and TUNEL assay.

**Additional file 8. **GSDMD shRNA. Identification of GSDMD as a required component for pyroptosis (GSDMD shRNA).

**Additional file 9. **LDH. Hydrogen treatment upregulated LDH release in endometrial cancer cells.

**Additional file 10.** ELISA. Hydrogen treatment upregulated IL-1β release by ELISA in endometrial cancer cells.

**Additional file 11.** in vivo. Hydrogen-rich water treatment inhibits endometrial tumorigenesis in vivo.

## Data Availability

All data generated or analyzed during this study are included in this published article in Additional files.

## Abbreviations

AN3CA-LUC cells: luciferase-AN3CA cells
ASC: Adaptor protein apoptosis associated speck-like protein containing ASC (a caspase recruitment domain, CARD)
DCFH-DA: Dichlorofluorescin diacetate
DMSO: Dimethyl suiphoxide
ELISA: Enzyme-linked immunosorbent assay
GSDMD: Gasdermin D
H2: Hydrogen
H-CM: Hydrogenated DMEM culture medium (H-CM)
IHC: immunohistochemical
LDH release: Lactate dehydrogenase release
NF-κB: Nuclear factor-κB
NLRP3: Nucleotide-binding domain (NOD)-like receptor (NLR) family member pyrin domain-containing protein 3
PI staining: Propidium iodide staining
PYD: clustered pyrin domains
ROS: Reactive oxygen species
RT-qPCR: Reverse transcription-quantitative polymerase chain reaction
shRNA: short hairpin RNA
siRNA: small interfering RNA
STR: Short Tandem Repeat
TMT: Tandem Mass Tag
TNF-α: Tumor necrosis factor
TUNEL assay: TdT-mediated dUTP Nick-End Labeling assay
