# Supplementary material for: Hydrogen inhibits endometrial cancer growth via a ROS/NLRP3/caspase-1/GSDMD-mediated pyroptotic pathway
Source: BMC Cancer. 2020 Jan 10;20:28. doi: 10.1186/s12885-019-6491-6 (PMC6954594; doi:10.1186/s12885-019-6491-6)
Supplement: Supplementary file 1 — Additional file 1. High-throughput sequencing in TCGA database. High-throughput sequencing of NF-κB pathway gene associated with GSDMD and NLRP3 in 176 endometrial cancer specimens via the TCGA database. [file 12885_2019_6491_MOESM1_ESM.docx]

**Supplementary Table 1**

| Gene/ P value | NFKB1 | NFKB2 | NFKBIA | NFKBIE | NFKBIL1 | NFKBIL2 |
| --- | --- | --- | --- | --- | --- | --- |
| NLRP3 | 6.28E-07 | 0.00092232 | 7.70E-07 | 6.07E-06 | 0.01198149 | 0.0009199 |
| GSDMD | 0.02517886 | 3.60E-12 | 0.0002133 | 3.13E-17 | 1.04E-11 | 0.00033431 |

High-throughput sequencing of NF-κB pathway gene associated with GSDMD and NLRP3 in 176 endometrial cancer specimens via the TCGA database.
