# Supplementary material for: Hydrogen inhibits endometrial cancer growth via a ROS/NLRP3/caspase-1/GSDMD-mediated pyroptotic pathway
Source: BMC Cancer. 2020 Jan 10;20:28. doi: 10.1186/s12885-019-6491-6 (PMC6954594; doi:10.1186/s12885-019-6491-6)
Supplement: Supplementary file 2 — Additional file 2. Significantly affected pathways and genes. Significantly affected pathways and genes in hydrogen-treated endometrial cancer cells. [file 12885_2019_6491_MOESM2_ESM.docx]

**Supplementary Table 2** Significantly affected pathways and genes in hydrogen-treated endometrial cancer cells.

|  |  | HEC1A | | | AN3CA | | |
| --- | --- | --- | --- | --- | --- | --- | --- |
| Pathway | TNF | 4.35E-09 |  |  | 0.001093492 |  |  |
|  | NF-κB | 2.90E-05 |  |  | 0.021789819 |  |  |
|  | Apoptosis | 0.032489587 |  |  | 0.003746339 |  |  |
|  | Necroptosis | 0.021919497 |  |  |  |  |  |
|  | Ferroptosis |  |  |  | 0.01172137 |  |  |
| Gene | GENE/FPKM | H-CM | CM | Log2FC | H-CM | CM | Log2FC |
|  | NFKBIA | 103.5338 | 20.9748 | 2.3034 | 11.5170 | 4.2156 | 1.4500 |
|  | NFKBID | 13.9361 | 1.1728 | 3.5708 |  |  |  |
|  | NFKBIE | 16.0616 | 5.5589 | 1.5307 |  |  |  |
|  | NFKBIZ | 19.8233 | 5.2468 | 1.9177 |  |  |  |
|  | TNF | 83.2268 | 4.4283 | 4.2322 |  |  |  |
|  | TNFFRSF12A |  |  |  | 72.5851 | 32.6434 | 1.1529 |
|  | TNFAIP2 | 89.2708 | 30.6025 | 1.5445 |  |  |  |
|  | TNFAIP3 | 8.7570 | 1.1613 | 2.9147 |  |  |  |
|  | TNFRSF21 | 17.6954 | 7.5398 | 1.2308 |  |  |  |
|  | JUN | 75.7223 | 12.9895 | 2.5434 | 22.3262 | 7.0776 | 1.4264 |
|  | JUNB | 210.8038 | 64.9215 | 1.6991 | 69.6526 | 31.2481 | 1.1564 |
|  | JUND | 24.9834 | 12.4495 | 1.0049 |  |  |  |

Genes in the signaling pathways of TNF, NF-κB, Apoptosis, Necroptosis and Ferroptosis were significantly upregulated. Both TNF and NF-kappa B signaling pathways in HEC1A and AN3CA cells were regulated by hydrogen treatment.
