# Supplementary material for: Hydrogen inhibits endometrial cancer growth via a ROS/NLRP3/caspase-1/GSDMD-mediated pyroptotic pathway
Source: BMC Cancer. 2020 Jan 10;20:28. doi: 10.1186/s12885-019-6491-6 (PMC6954594; doi:10.1186/s12885-019-6491-6)
Supplement: Supplementary file 3 — Additional file 3. TCGA data. NLRP3, caspase-1, and GSDMD protein expression in endometrial cancer related to survival rate based on the TCGA data. [file 12885_2019_6491_MOESM3_ESM.docx]

**NLRP3, caspase-1, and GSDMD protein expression in endometrial cancer related to survival rate based on the TCGA data:**

**Table:**

|  |  |  |  | **Histological subtypes** |  |
| --- | --- | --- | --- | --- | --- |
|  | **Comparison** | **Statistical significance** |  | **Comparison** | **Statistical significance** |
| **NLRP3** | **Normal-vs-Primary** | **2.46E-07** |  | **Normal-vs-Endometrioid** | **7.88E-05** |
| **CASP1** | **Normal-vs-Primary** | **3.95E-01** |  | **Normal-vs-Endometrioid** | **3.89E-01** |
| **GSDMD** | **Normal-vs-Primary** | **5.52E-07** |  | **Normal-vs-Endometrioid** | **2.26E-06** |

**Figure:**

**
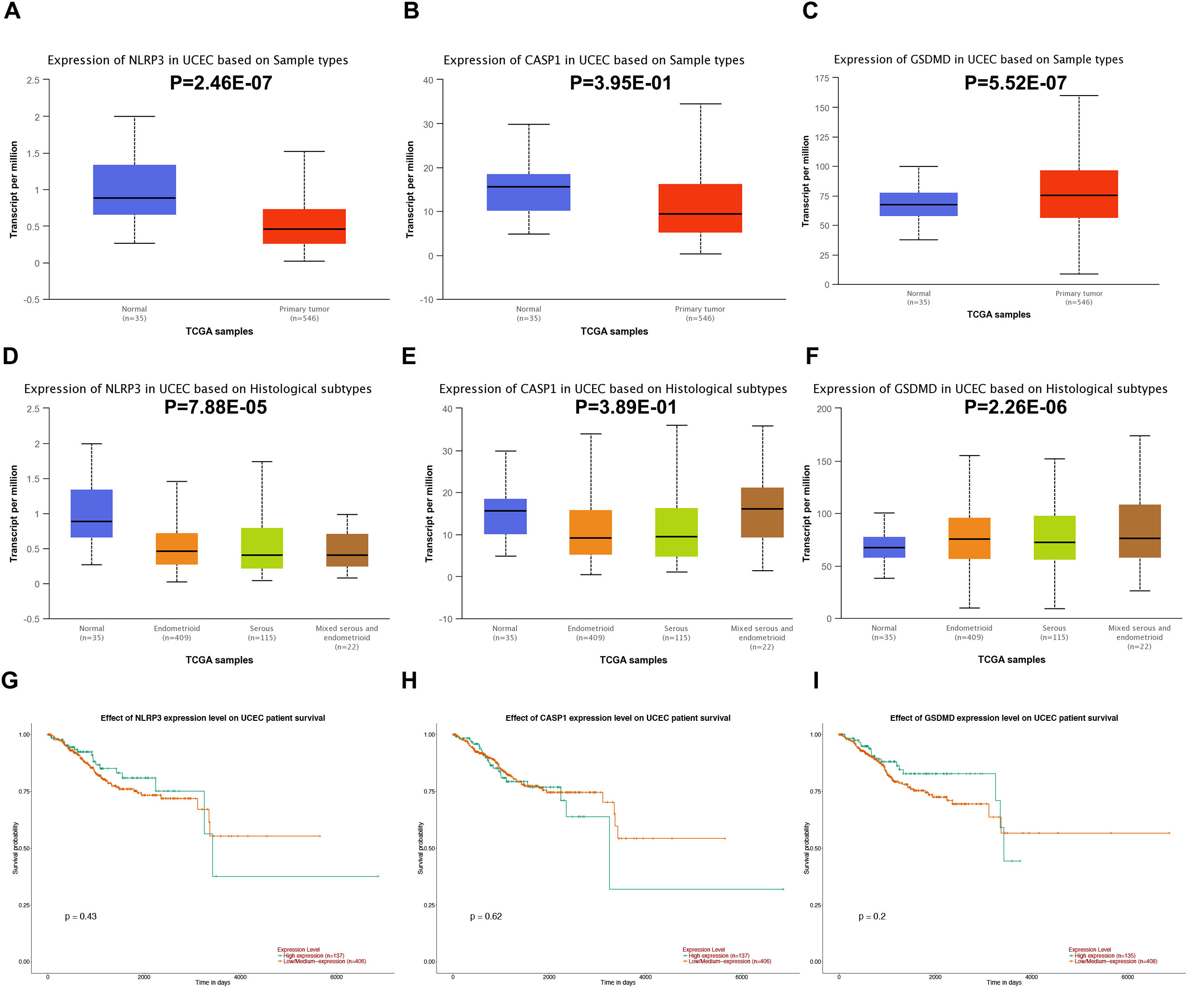
**

**Figure Legends**

From TCGA database. NLRP3 (**A.**) (P=2.46E-07) and GSDMD (**C.**) (P=5.52E-07) protein expression was significantly different between endometrial cancer and normal endometrium tissues. **B.** Expression of CASP1 in endometrial cancer was higher than that in normal endometrial tissues (P=3.95E-01). NLRP3 (**D.**) (P=7.88E-05) and GSDMD (**F.**) (P=2.26E-06) protein expression was significantly different between histological subtypes of endometrial cancer. **E.** Expression of CASP1 was different between different histological subtypes in endometrial cancer (P=3.89E-01).There was a trend of difference in NLRP3 (**G.**) (P=0.43), caspase-1 (**H.**) (P=0.62), and GSDMD (**I.**) (P=0.2) protein between high and low/medium expression group in endometrial cancer related to survival rate. Primary tumor (N=546): endometrial cancer; Normal (N=35): normal endometrium; CASP1: caspase-1
